# Lymphopenia as a predictor of bacteremia in the emergency department

**DOI:** 10.1186/cc13410

**Published:** 2014-03-17

**Authors:** R Lowsby, C Gomes, I Jarman, P Nee

**Affiliations:** 1 St Helens and Knowsley Teaching Hospital NHS Trust, Liverpool, UK; 2 Liverpool John Moores University, Liverpool, UK

## Introduction

Bloodstream infection (BSI) is associated with a reduction in circulating lymphocytes. Lymphopenia has been proposed as an early marker of BSl in pyrexial adults in the emergency department (ED) setting [1]. The aim of this study was to compare lymphocyte count with conventional markers in patients presenting to the ED of a UK hospital with suspected sepsis.

## Methods

A retrospective review was undertaken of adult patients presenting to the ED (annual census 90,000) with pyrexial illness over a 12-month period, 2011 to 2012. Data included white cell count (WCC), neutrophil count (NC), lymphocyte count (LC) and C-reactive protein (CRP). The results were compared. Sensitivity and specificity were calculated for each parameter and receiver operating characteristic (ROC) curves were constructed.

## Results

**Figure 1:**
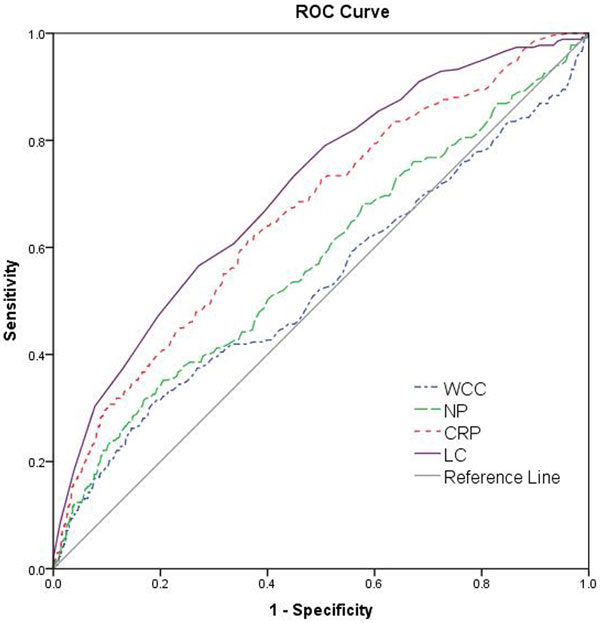
**ROC curve comparing blood test parameters in predicting bacteremia**.

A total of 2,515 patient records were screened. Patients on chemotherapy were excluded, as were those under 18 years old. In total, 1,954 patients (53% female, median age 66 years) were included in the analysis. Blood cultures were positive in 13.7% of cases. There were significant differences between all variables measured, with the exception of WCC, between bacteremic and nonbacteremic patients. The area under the curve of 70.8 was best for lymphocyte count (Figure 1).

## Conclusion

In adult patients presenting to the ED with pyrexial illness, lymphopenia predicts bacteremia better than the usual markers of infection.

## References

[B1] deJager Crit Care 2010 14 R192 10.1186/cc9309 21034463 PMC3219299

